# Radiation-Induced Transient Currents in Films of Poly(arylene ether ketone) Including Phthalide Moiety

**DOI:** 10.3390/polym12010013

**Published:** 2019-12-19

**Authors:** Evgenii D. Pozhidaev, Vera V. Shaposhnikova, Alexey R. Tameev, Andrey E. Abrameshin

**Affiliations:** 1 National Research University Higher School of Economics, 101000 Moscow, Russia; tameev@elchem.ac.ru (A.R.T.); aabrameshin@hse.ru (A.E.A.); 2 Nesmeyanov Institute of Organoelement Compounds of the Russian Academy of Sciences, 119991 Moscow, Russia; vsh@ineos.ac.ru; 3 Frumkin Institute of Physical Chemistry and Electrochemistry of the Russian Academy of Sciences, 119071 Moscow, Russia

**Keywords:** co-polymer, polyarylene ether ketones, switching effect, irradiation by electrons, small-signal regime, radiation induced conductivity, dispersive transport, carrier mobility

## Abstract

The electrical properties of thin films of poly(arylene ether ketone) copolymers (co-PAEKs) with a fraction of phthalide-containing units of 3, 5, and 50 mol% in the main chain were investigated by using radiation-induced conductivity (RIC) measurements. Transient current signals and current-voltage (*I-V*) characteristics were obtained by exposing 20 ÷ 25 μm thick films of the co-PAEKs to monoenergetic electron pulses with energy ranging from 3 to 50 keV in an electric field ranging from 5 to 40 V/μm. The Rose-Fowler-Vaisberg semi-empirical model based on a multiple trapping formalism was used for an analysis of the RIC data, and the parameters of the highly dispersive charge carrier transport were evaluated. The analysis revealed that charge carriers moved in isolation from each other, and the applied electric fields were below the threshold field triggering the switching effect (a reversible high-to-low resistivity transition) in the co-PAEK films. It was also found that the co-PAEK films, due to the super-linear *I*-*V* characteristics, are highly resistant to electrostatic discharges arising from the effects of ionizing radiation. This property is important for the development of protective coatings for electronic devices.

## 1. Introduction

Intensive researches on the use of polymers as microelectronics elements are under way [1,2]. Thin films of polydiphenylterephthalide (PDP) are known to exhibit reversible electroresistive state switching (ERSS) called switching effect [1,3]. In a strong electric field, the switching effect consists in a sharp jump reversible transition of the polymer resistivity from a high-ohmic state to a low-ohmic state. ERSS is important for the development of next-generation non-volatile memory devices. A polymer material with intrinsic switching effect would be of great interest because its properties can be adjusted over a wide range by fabrication techniques and modifications of the molecule structure.

The origin of the resistivity switching in the PDP thin films is explained by the transition of the conformation of the macromolecules between two stable states, due to the reorganization of the phthalide moieties by the action of an external electric field [1]. At a high resistance state, the charge carrier mobility in PDP films was of the orders of 10^−5^ and 10^−4^ cm^2^ V^−1^ s^−1^, with a tendency to increase as the electric field approached the threshold switching voltage to the low resistance state [4]. Similar charge mobility values were also demonstrated by a polyimide-consisting phthalide moiety in the polymer unit [5].

There is still no generally accepted physical model describing the switching effect in thin polymer films. This complicates the productive development of microelectronic devices, although the low conduction switching thresholds in electric fields and the reversibility of these effects seem highly promising. A practically important field of application of such polymeric materials is obviously the protective coating of electronic devices. Film coating made of polymeric material that is capable of transporting charge carriers prevents the accumulation of charge when subjected to electron or ionizing radiation and thereby prevents subsequent electrostatic discharges leading to failures of the electronic device.

Poly arylene ether ketones (PAEKs) possess reasonable electrophysical properties, along with high thermal, chemical, and mechanical resistance [6,7,8,9,10]. Thin films of PAEK co-polymers, as well as the PDP thin films, exhibit reversible ERSS [1,11]. Therefore, by varying the phthalide content of co-PAEKs, one can tune the electrical properties of the polymer films [11]. In this regard, a study of electrical conductivity in co-PAEKs with various contents of phthalide fragments in the units is of particular interest for revealing the resistivity switching ability in films of the order of ten microns in thickness.

This article presents the results of the electronic transport investigation in films of co-PAEK with various contents of phthalide moiety, using the enhanced radiation-induced conductivity (RIC) measurement method combined with the time-of-flight technique (TOF) [12]. In this method, two types of charge introduction into the test sample are implemented: near the front electrode (as for the conventional TOF) and in the bulk of the polymer film (RIC). Exposing polymer films to monoenergetic electron pulses of energy ranging from 3 to 50 keV allowed us to investigate tens of microns thick films. The experimental approach for investigating the transport of charge carriers in the co-PAEK films is of particular interest in detecting the resistance of the co-polymer to electrostatic discharges due to ionizing radiation.

## 2. Experimental Details

### 2.1. Materials

**Figure 1:**
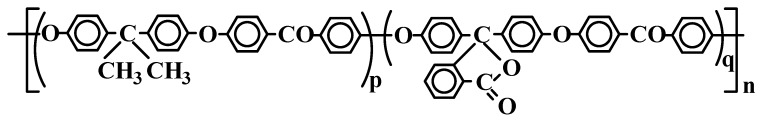
Phthalide moiety containing co-poly(arylene ether ketones) (co-PAEKs) with the p/n ratio ranged as 0.97/0.03, 0.95/0.05, and 0.5/0.5.

The co-PAEKs shown in Figure 1 were synthesized by polycondensation of 4,4′-difluorobenzophenone with dipotassium bisphenolates proceeding from the mechanism of nucleophilic substitution of activated halogen in the aryl dihalide, analogously to the earlier described preparation of homopolymers [13]. The concentration of the bisphenols mixture was 0.5 mol per 1 L of the solvent, with a 30% excess of K_2_CO_3_. As an example, the procedure for the synthesis of co-PAEK (with a copolymer unit ratio of p:n = 0.5:0.5) based on 2,2-bis(4′-hydroxyphenyl)propane, 3,3-bis(4′-hydroxyphenyl)phthalide, and 4,4′-difluorobenzophenone was as follows: 4,4′-difluorobenzophenone (0.1 mol), 2,2-bis(4′-hydroxyphenyl)propane (0.05 mol), and 3,3-bis(4′-hydroxyphenyl)phthalide (0.05 mol) premilled freshly, and calcined K_2_CO_3_ (0.13 mol), DMAA (200 mL), and chlorobenzene (100 mL) were loaded in an argon-blown four-necked flask equipped with a stirrer, an argon supply tube, and a system for azeotropic removal of water. The flask was heated in an oil bath whose temperature was increased from 25 °C up to 185 °C within ~0.5 h. After the complete removal of an azeotropic chlorobenzene–water mixture, the synthesis lasted 7 h. The reaction mixture was cooled and dissolved in chloroform. The resulting solution was filtered from salts and washed by stirring with water many times. After the evaporation of the chloroform solution at 25 °C and drying by gradually increasing the temperature from 60 to 140 °C over a period of 18 h, and then at 160 °C for 25 h, the co-PAEK was obtained as a film with a yield of 99%. The reduced viscosity (*η*_red_) determined for a solution of the polymer in chloroform (0.5 g/100 mL) at 25 °C was 0.50 dL g^−1^. The other synthesized co-PAEKs possessed a high molecular weight and *η*_red_ = 0.54–0.82 dL g^−1^.

All the polymers are readily soluble in a wide range of solvents (dichloromethane, chloroform, sym-tetrachloroethane, THF, dioxane, cyclohexanone, m-cresol, DMF, DMAA, etc.); their films formed by drop cast from a solution are strong (the tensile strength was 77–85 MPa) and transparent in the spectral range of 400–1200 nm. In the studied co-PAEKs, the fraction of phthalide-containing units was n = 3, 5 and 50 mol%.

4,4′-Difluorobenzophenone, 2,2-bis(4′-hydroxyphenyl)propane, 3,3-bis(4′-hydroxyphenyl)phthalide, *N*,*N*-Dimethylacetamide (DMAA), chlorobenzene, potassium carbonate, and other chemicals with all in analytical grade were purchased from Acros Organics Co. Ltd. (Moscow, Russia).

4,4′-Difluorobenzophenone was recrystallized from ethanol before use. Anhydrous K2CO3 was ground by mortar and pestle and dried at 1300C for 3 h before use. 2,2-Bis(4′-hydroxyphenyl)propane, 3,3-bis(4′-hydroxyphenyl)phthalide, *N*,*N*-dimethylacetamide (DMAA), chlorobenzene were used as received without further purification.

### 2.2. Methods

**Figure 2:**
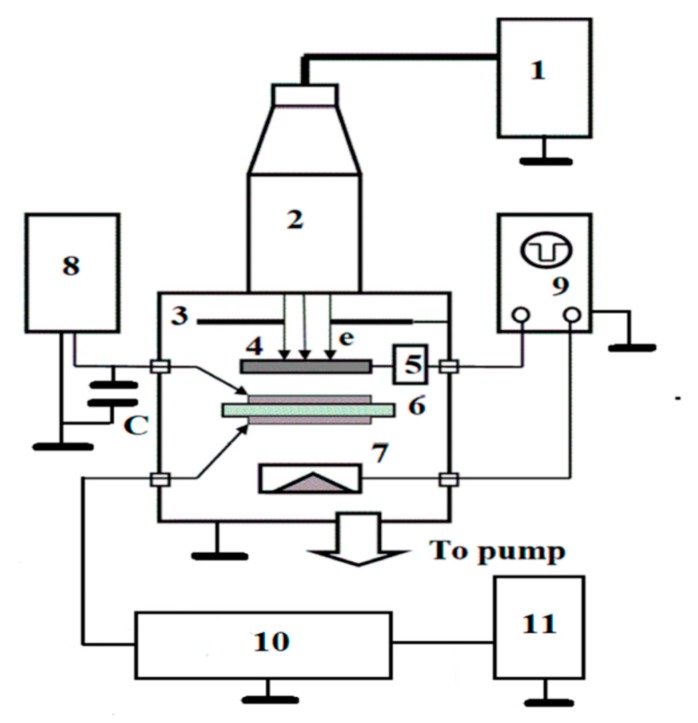
Scheme of the experimental setup for measuring polymer radiation-induced conductivity (RIC) and the time-of-flight (TOF) measurement. 1—high-voltage power supply; 2—electron gun; 3—electron beam collimator; 4—metallic shutter; 5—shutter control system; 6—test sample with evaporated Al electrodes; 7—Faraday cup; 8—DC voltage supply with an accumulative capacitor C and an electric circuit to turn the output voltage on and off and to control it; 9—double-beam Tektronix 3012B oscilloscope with a bandwidth of 300 MHz; 10—electronic block for measuring an analog RIC signal, amplifying and analog-to-digital converting, and finally sending the ORIGIN current curve to printer 11.

RIC and TOF measurements: a scheme of the experimental setup for measuring polymer radiation-induced conductivity (RIC) and the time-of-flight (TOF) measurement is given on Figure 2 and detailed in [14].

The most valuable information has been obtained using 50 keV electrons (maximum range of about 40 μm) in pulse (1 ms) and continuous (exceeding approximately 0.1 s) irradiation modes, while the TOF application (electron energy 3–7 keV with a range less than 1.5 μm) was unsuccessful due to co-PAEK electronic properties.

RIC irradiations of 50 keV electrons were normally incident upon polymer samples inside a vacuum chamber with the ELA-65 electron gun at room temperature only. The dose rate depth profile was typical for 50 keV electrons, so that an average dose rate was estimated to be 2 times larger than at the front surface of a sample. The RC time constant was about 1 ms.

All 20 to 25 μm thick (40 mm in diameter) specimens were supplied with evaporated 50 nm thick Al electrodes with a diameter of 32 mm. The applied electric field ranged from 20 to 40 V/μm. We used only just-prepared samples for each experimental run. RIC and TOF techniques have already been described in detail [15,16].

## 3. Results

**Figure 3:**
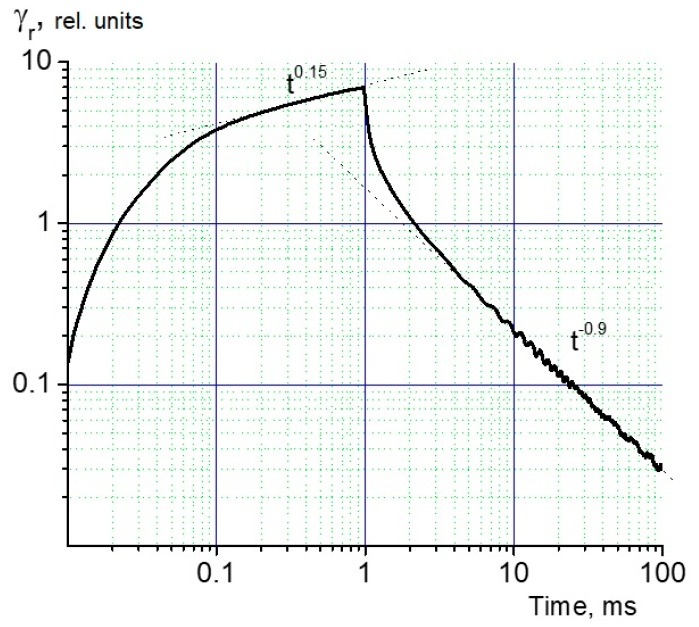
RIC current transient recorded in a small signal regime. Pulse length 1 ms, electric field 40 V/μm (a flat pulse top begins after 0.1 ms). Co-PAEK with the p/n ratio of 0.95/0.05.

We start with the presentation of the RIC pulse results, which are most straightforward (Figure 3). The pulse length was 1 ms and the flat top of the pulse extended from 100 s to the pulse end (the RC time constant was 10 s). The irradiation was conducted in a small signal regime with no recombination present at any observation time.

The most important information from Figure 3 is as follows: the maximum RIC happens at the pulse end and is equal to $K_{rm}$ = 1.7 × 10^−14^ Ω^−1^ m^−1^/(Gy/s) ≡ F·m^−1^·Gy^−1^ (the RIC per unit dose rate), which is a legitimate characteristic of a polymer at a small signal irradiation. It was seen that RIC rose with time, slightly slowing down to the pulse end when its logarithmic slope β=dlgγ/dlgt became equal to 0.15. After the pulse end, the RIC fell rapidly during the initial 500 s but then stabilized at a falling rate $\beta_{f}=-\beta$, in our case = 0.95.

We could see no traces of the transit time effects (the fact that $\beta_{f}$ was close to a unity strongly hampered their observation (see below)). By the way, the $K_{rm}$ in PAEK happened to be 9 times smaller than that of polystyrene, in which the transit time effects were also absent at this high field.

It could be seen that at this pulse length the delayed component of RIC ($\gamma_{rd}$) coexisting with the prompt ($\gamma_{rp}$) clearly dominated, so that it did not exceed 0.4 × 10^−14^ F·m^−1^·Gy^−1^.

**Figure 4:**
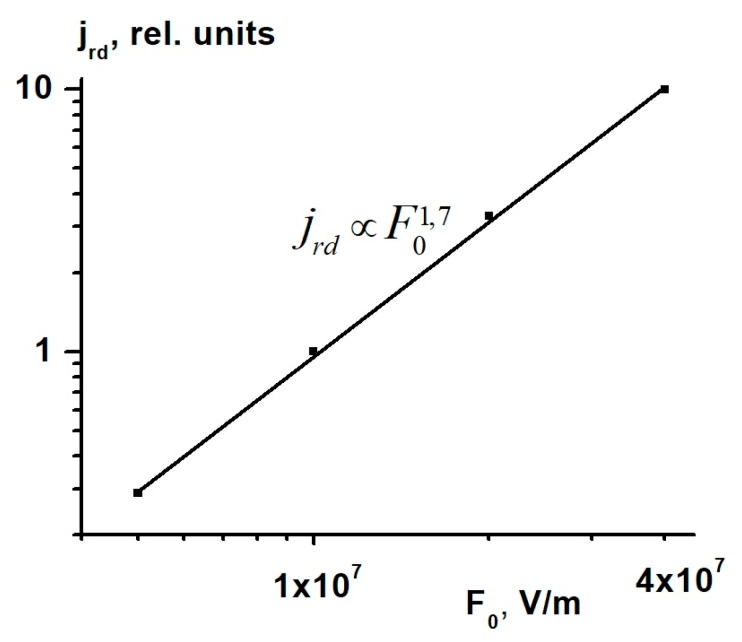
The current-voltage (*I-V*) characteristic of co-PAEK films with the p/n ratio of 0.5/0.5.

Figure 4 shows the *I-V* characteristic of co-PAEK films with the p/n ratio of 0.5/0.5. The current of the radiation conductivity delayed component in relative units was measured along the ordinate axis. We see that in contrast to $K_{rp}$, the delayed component current and $K_{rd}$ strongly depend on the applied electric field *F*_0_. In the field range 5 to 40 V/μm, $K_{rd}\infty F^{1.7}$, thus testifying to the Onsager mechanism of the free charge carrier generation [17].

An attempt to study charge carrier mobility directly using the TOF technique, as we did earlier with molecularly doped polymers, failed, as Figure 3 shows: the current curve simply duplicated the respective RIC curves, producing no kinks on the current transients.

**Figure 5:**
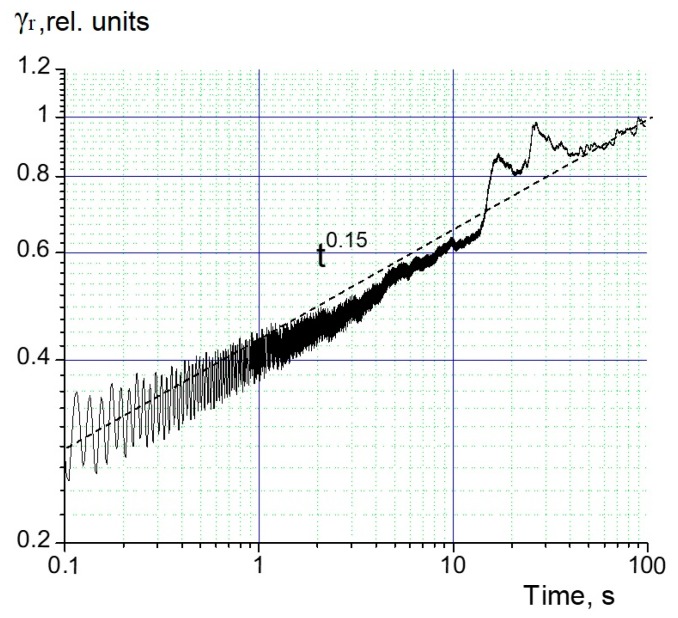
RIC transient current taken in a small signal irradiation. Dose rate 19 Gy/s, electric field 40 V/m, irradiation time 100 s. The RIC rise is approximated with a dashed straight line with the same slope (0.15) as the pulse irradiation at 1 ms (the transient curve is slightly distorted by radio frequency noise (rf-noise) and the beam current instability).

In principle, the above information suffices to construct a mobility model in co-PAEK (see below), but to extend it to longer times, we supplemented our pulse RIC measurements with the long-term irradiations still in the small signal regime (Figure 5).

In this figure, the RIC rise is approximated with a dashed straight line with the slope β=0.15. The transient curve was slightly distorted by rf-noise, and two current releases in the range of 10–50 s were related to the beam current instability caused by the interference in the power supply circuit.

It is important that the RIC showed the same pattern of a rising current, as observed during 1 ms pulse irradiation (slopes β were the same). Moreover, the extrapolation of the RIC from 1 ms through the dead time gap to 0.1 s (opening time of a shutter), when an irradiation with a constant dose rate was established (2.8 × 10^−14^ F·m^−1^·Gy^−1^), almost coincided with the measured one at 0.1 s (3.1 × 10^−14^ F·m^−1^·Gy^−1^), well within error bars (20%). It follows that the delayed RIC rose according to the power law $\gamma_{rd}\infty t^{0.15}$ in a broad time range of up to 100 s. As we will see below, this fact allows for a definitive conclusion about charge carrier transport in PAEK.

## 4. Discussion

To analyze the above RIC data, we used the Rose-Fowler-Vaisberg (RFV) semi-empirical model based on a multiple trapping formalism [18,19]. The basic equations of the conventional one-carrier RFV model are as follows:

(1) $$\{\begin{array}{l} \frac{dN}{dt}=g_{0}-k_{r}N_{0}N,\\ \frac{\partial \rho }{\partial t}=k_{c}N_{0}\left[\frac{M_{0}}{E_{1}}\exp\left(-\frac{E}{E_{1}}\right)-\rho \right]-\nu_{0}exp\left(-\frac{E}{kT}\right)\rho \\ N=N_{0}+\int_{0}^{\infty }\rho dE. \end{array},$$

At *t* = 0, both *N*_0_(*t*) and *ρ*(*E*,*t*) are equal to 0.

By definition, the RIC is $\gamma_{r}(t)=e\mu_{0}N_{0}(t)$. Thus, system (2) refers to unipolar (by tradition, electron) conduction. Here, *N*(t) is the total concentration of radiation-produced electrons (equal to that of the holes); $N_{0}(t)$ is the concentration of mobile electrons in extended states (in transfer band) with microscopic mobility $\mu_{0}$; $g_{0}$ is the generation rate of free charge carriers (assumed time and space independent during irradiation); $k_{r}$ is the recombination coefficient of mobile electrons with immobile holes acting as recombination centers; $k_{c}$ is the trapping rate constant; $M_{0}$ is the total concentration of traps exponentially distributed in energy E, which is positive and taken downwards from the energy level of the transport band; $E_{1}$ is the parameter of the trap distribution; ρ(E,t) is the time-dependent density distribution of trapped electrons; $\nu_{0}$ is the frequency factor; T is the temperature; k is the Boltzmann’s constant; and e is an elementary electric charge. The dispersive parameter α, which defines the major temporal features of the transient curves, is equal to $kT/E_{1}$. Also, $\tau_{0}={\left(k_{c}M_{0}\right)}^{-1}$ is the lifetime of mobile electrons before trapping. Of course, $g_{0}$ is proportional to the dose rate $R_{0}$, depending on the temperature and an applied electric field. In the case of hole-conducting polymers, the roles of electrons and holes are to be swapped.

In a small signal regime, Equation (1)’s simplification as a recombination term in the first equation may be neglected. Now, the RFV model allows analytical solutions showing that the logarithmic slope *β* coincides with the dispersion parameter *α* of the model. Moreover, theory shows that the following relationship holds (α ≤ 0.5, $\nu_{0}t\gg 1$):

(2) $$K_{rd}(t)\approx \eta_{0}\mu_{0}\tau_{0}e{\left(\nu_{0}t\right)}^{\alpha }.$$

Here, *η*_0_ = *g*_0_/*R*_0_ and *η*_0_*e* = 10*ρG_fi_*, where *ρ* is polymer density (g/cm^3^) and *G_fi_* is the yield of free charge carriers per 100 eV of absorbed energy. According to the Onsager mechanism, this quantity is close to 0.8 at 40 V/μm and room temperature. Taking *ρ* ≈ 1.2, we immediately recover the triple product *P* = *μ*_0_*τ*_0_(*ν*_0_)^α^ that defines the expected time of flight through polymer film (L is its thickness):

(3) $$t_{dr}\approx {\left(L/PF_{0}\right)}^{1/\alpha }.$$

In our case, 1/α ≈ 6.67, so that taking *t* = 0.1 s and *K*_rd_ = 3.1 × 10^−14^ F m^−1^ Gy^−1^, we find that *P* = 4.4 × 10^−14^ (in system SI). As a result, an expected time of flight through the 20 μm thick co-PAEK film at an applied field *F*_0_ = 40 V/μm would be astronomically large (10^13^ s). No wonder that our TOF experiment failed. Reducing the sample thickness to 1 μm makes the transit time much shorter (2 × 10^4^ s) but still too large. In fact, one can speak about transit time effects only in polymer layers thinner than 0.1 μm.

Thus, in about 20 μm thick films at electric fields of 5–40 V/μm, the charge carrier transport is highly dispersive, and the carriers exhibit low mobility. The received results indicate that in the studied range of electric field the increase in quantity of the co-PAEK units with the phthalide moiety from the p/n ratio of 0.97/0.03 to 0.5/0.5 did not lead to a noticeable RIC change within an experimental error. Therefore, in our experiments, charge carriers moved in isolation from each other at electric fields, which were still insufficient for triggering the switching. Indeed, ultrathin films withstand electric fields of up to 10^8^ V/m. Such electric fields were not achievable in our experiments because a 20 μm polymer film underwent electrical breakdown. The very low mobility may have been caused by geometric constraints associated with the arrangement of phthalide groups in the co-PAEK. The distance between the charge carriers within the phthalide monomer unit was much smaller than that between phthalide groups separated by an arylene monomer unit *p* (Figure 1). Thus, the hopping of charge carriers between phthalide groups serving as charge transporting sites was difficult.

This consideration is consistent with the mechanism, suggesting that the dielectric-to-conductive state switching occurs due to the formation of conductive channels in a polymer film [20]. In the RIC experiments at a low electric field, the lifetime of generated charge carriers (i.e., charge pairs (CPs)) was short because of their easy geminate recombination. At a high electric field, those CPs can be in a metastable energy state, and the recombination remarkably decreases, as evidenced by the experiment on delayed luminescence in a conjugated polymer [21]. This leads to an increase in the concentration of CPs, needed for a strong overlap of the Coulomb potentials and for providing the high charge mobility within the conductive channels.

Another point is related to the films reaching a pre-threshold state time. The co-PAEK films (p/n = 0.5/0.5) were exploited in silicon solar cells as a transparent conductive polymer [11]. Yet, in our RIC measurements, the co-PAEK films did not switch from dielectric to conductive state during the observed transient current. Thus, one may conclude that the switching of these co-PAEK films can occur for a much longer time than the duration of the transient RIC experiments.

Besides, the non-linearity of the *I-V* characteristics of the co-PAEK films shows that, at strong pre-breakdown fields, an increase of the super-linearity can be expected. In this condition, when the C–O bond in the phthalide group is broken [1], a transition from the transport of isolated (individual) charge carriers, which was observed in our experiments, to a collective interaction with the above-mentioned conductive channel formation [20] can occur. In RIC experiments, charge carriers move in isolation, whereas collective movements are observed in the switching effect. Therefore, we just started with the measurement of the charge carrier mobility. In order to continue the switching effect research in thin films of electroactive polymers, further setting of the experiment is necessary.

The increased stability of the co-PAEK films when exposed to ionizing radiation and their super-linear *I-V* curves results in their increased resistance to electrostatic discharges. As seen in Figure 4, as the electric field increases eight times, the current flowing through the sample and characterizing Maxwell’s relaxation time of the injected charge increases more than 30 times, which dramatically reduces the probability of reaching breakdown fields.

## 5. Conclusions

In this work, we have used the enhanced radiation-induced conductivity (RIC) measurement method combined with the time-of-flight technique (TOF) in order to investigate the electron transport in films of co-polymers of poly arylene ether ketones (co-PAEKs). For the study, a number of co-PAEKs differing in the content of phthalide-containing units in the main chain (3, 5, and 50 mol%) have been synthesized, and their 20 to 25 μm thick films have been prepared.

In the electric field *F*, ranging from 5 to 40 V/μm, a strong non-linearity (super-linearity) of current-voltage (*I*-*V*) characteristics with the relation $K_{rd}\infty F^{1.7}$ has been observed in the co-PAEK films. Such *I*-*V* characteristics indicate that the free charge carrier generation obeys the Onsager mechanism.

The RIC measurements have shown that the charge carrier transport is strongly dispersive, with the low dispersive parameter α = 0.15. The analysis of the experimental data in terms of the Rose-Fowler-Vaisberg model has revealed that transit time effects on the parameter are to be expected only in very thin polymer layers (less than 0.1 μm in thickness), even in strong electric fields of about 40 V/μm.

Within the used range of electric field, the increase in the concentration of phthalide-containing units in co-PAEKs from 3 to 50 mol% has not influenced the data on RIC in all the co-PAEKs. In the finding points used in our experiments charge carriers moved in isolation from each other, and the range of the applied electric field was insufficient for triggering the switching effect in the co-PAEK films.

The type of ionizing radiation we have used affects the electronic devices of spacecrafts operating in geostationary and highly elliptical orbits, especially during geomagnetic perturbations [22,23]. We have shown that the co-PAEK films, due to their super-linear *I*-*V* characteristics, possess increased resistance to electrostatic discharges arising from the effects of ionizing radiation.
